# The role of regulatory B cells in immune regulation and childhood allergic asthma

**DOI:** 10.1186/s40348-023-00174-2

**Published:** 2024-01-04

**Authors:** Caroline Vanessa Kliem, Bianca Schaub

**Affiliations:** 1 Pediatric Allergology, Department of Pediatrics, Dr. Von Hauner Children´S Hospital, University Hospital, Lindwurmstraße 4, 80337, LMU Munich, Germany; 2 Member of German Center for Lung Research - DZL, LMU, Munich, Germany; 3 Member of German Center for Child and Adolescent Health–DZKJ, LMU, Munich, Germany

**Keywords:** Allergic asthma, Childhood asthma, B cells, Regulatory B cells, Immune system

## Abstract

**Background:**

As the most common chronic disease in childhood, asthma displays a major public health problem worldwide with the incidence of those affected rising. As there is currently no cure for allergic asthma, it is mandatory to get a better understanding of the underlying molecular mechanism.

**Main body:**

By producing IgE antibodies upon allergen contact, B cells play a pivotal role in allergic asthma. Besides that, IL-10-secreting B cell subsets, namely regulatory B cells (Bregs), are reported in mice and humans to play a role in allergic asthma. In humans, several Breg subsets with distinct phenotypic and functional properties are identified among B cells at different maturational and differentiation stages that exert anti-inflammatory functions by expressing several suppressor molecules. Emerging research has focused on the role of Bregs in allergic asthma as well as their role for future diagnostic and preventive strategies.

**Conclusion:**

Knowledge about the exact function of human Bregs in allergic asthma is still very limited. This review aims to summarize the current knowledge on Bregs. We discuss different human Breg subsets, several ways of Breg induction as well as the mechanisms through which they exert immunoregulatory functions, and their role in (childhood) allergic asthma.

## Background

Asthma is an inflammatory airway disease, affecting both children and adults, being the most common chronic disease in childhood. It is characterized by airway hyperresponsiveness, acute and chronic bronchoconstriction, airway edema, and mucus plugging [1]. There are two main forms of asthma: allergic and non-allergic asthma [2]. The World Health Organization (WHO) estimated that 262 million people were affected by asthma in 2019 [3], and with current trends rising, it is expected to reach 400 million by 2025 [4]. To this end, understanding the underlying disease mechanisms for further treatment is of great importance. Currently, there is no cure for asthma and therapy, which is focused on inhaled corticosteroids and bronchodilators, suppresses symptoms rather than changing the natural history of the disease [5]. Additional asthma therapies, also approved for children, use biologicals such as Omalizumab or Dupilumab which target IgE or asthma-associated cytokines such as IL-4 and IL-13, respectively [6]. Nevertheless, depending on the severity, asthma results in a diminished quality of life and an important economic burden on public health care systems, thus representing a major public health problem worldwide with a high socio-economic impact [7].

The immune system has several mechanisms to defend itself against viral, bacterial, fungal, and protozoal infections. For this, the mammalian immune system consists of the innate and the adaptive immune system. The latter is highly specific and relies on diverse antigen-specific receptors expressed on the surface of T- and B-lymphocytes [8]. For healthy immune regulation and to avoid excessive responses, the immune system needs to be tolerant against self and external innocuous antigens, referred to as immune tolerance. In healthy individuals, there is a subtle balance between anti-inflammatory (prevention of chronic inflammation and tissue damage) and pro-inflammatory (counter infections) responses [9]. Dysregulation can result in asthma, allergy, and autoimmune diseases [10].

The peripheral B cell compartment comprises a heterogeneous population of cells at different maturational stages along the lineage, each with distinctive functional properties [11]. They produce antibodies, cytokines, and act as antigen-presenting cells (APCs). In addition, B cells have the capability to regulate immune responses through regulatory B cells (Bregs), which are B cells with anti-inflammatory functions [12]. They exhibit their anti-inflammatory role by producing mainly anti-inflammatory cytokines, e.g., IL-10, transforming growth factor (TGF)-β, and IL-35, as well as by induction of other regulatory cells [13, 14]. In humans, Bregs are found among B-lymphocytes at different stages of maturation and differentiation, ranging from early transitional B cells to highly differentiated plasmablasts [15, 16].

Knowledge about the impact of B cells and Bregs on asthma is still rather limited. IgE production upon allergen contact is the main described role of B cells in allergic asthma, which is mandatory for the initiation of the allergic cascade [17]. Habener et al. revealed that regulatory B cells control airway hyperresponsiveness and airway remodeling in a murine asthma model, suggesting a potential role for B cells in future diagnostic and preventive strategies in asthma [18]. They also showed differences in human B cell populations between asthma and controls as well as between mild-moderate and severe asthma [19]. Moreover, Breg numbers contribute to the regulation of the immune system, which was confirmed by multiple human studies showing lower frequencies of some Breg subsets in allergic asthmatics [20–23]. This review aims to summarize the current knowledge about Bregs and their role in childhood allergic asthma to get new mechanistic insights into childhood asthma development and therapeutic and preventive strategies.

### Immune mechanisms underlying childhood allergic asthma

Allergic asthma is the predominant form of asthma in childhood, which has been characterized by sensitization to specific allergens, high IgE levels, eosinophilia, a type 2 shifted immune response, and decreased innate immunity gene expression [24]. Both the innate and the adaptive immune system play a role in type 2 immune response and involve several immune cells such as T-helper (Th) 2 cells, group 2 innate lymphoid cells (ILCs), B cells, natural killer (NK) cells, NK T cells, basophils, eosinophils, and mast cells, as well as their major cytokines [25].

Repetitive exposure of children to environmental allergens such as house dust mites (HDM) results in the development of allergen-specific memory T and B cell responses following expression of inflammatory mediators to recruit immune effector cells such as T and B cells [26, 27]. Upon allergen presentation by APCs to naïve CD4^+^ T cells, they get activated and release type 2 cytokines such as IL-3 and IL-5, which drive the proliferation and differentiation of basophils, eosinophils, and mast cells, which in turn contribute to asthmatic bronchial hyperresponsiveness. In addition, IL-4 and IL-13 are released, which play a key role in IgE production of B cells. IgE in turn binds to high-affinity IgE surface receptors (FcεRI) on immune cells such as dendritic cells (DCs), basophils, and mast cells [28, 29]. Upon cross-linking of the IgE-FcεRI complexes by the allergen, it activates the cells resulting in the release of inflammatory mediators like histamine, leukotrienes, and the type 2 cytokines IL-4, IL-5, and IL-13. Consequently, an influx of CD4^+^ T cells and eosinophils into the airways maintains type 2 inflammation. To this end, higher levels of lymphocytes, eosinophils, basophils, and mast cells are found in the airways of allergic asthmatics [28, 30]. Moreover, higher levels of airway epithelial desquamation, goblet cell hyperplasia, and thicker basement membranes are characteristics of asthmatics [29]. Since sensitization to allergens plays a key role in the development of allergic asthma [31], targeting of allergen sensitization seems to be promising in allergic asthma therapy [32].

### B cells

As part of the adaptive immune system, B cells play a pivotal role in the protection against pathogens. Moreover, they play important roles in several diseases such as autoimmune disorders [33, 34], cancer [35], allergy [36], and asthma [37, 38].

The peripheral B cell compartment consists of several cells at different maturational stages with different functions [11]. In humans, B cells develop in the bone marrow from hematopoietic precursors derived from the fetal liver [39]. Early B cell development includes (early and late) pro-B, pre-B, and immature B cells in which specific surface markers are expressed and are characterized by rearrangements of the immunoglobulin (Ig) heavy and light gene segments to generate diverse antigen receptors [40]. Immature B cells then exist in the bone marrow for several days until they enter the circulation as transitional B cells [41]. Depending on their surface markers and function, transitional B cells can be subdivided into T1, T2, and T3 B cells [42]. Depending on signals received through the B cell receptor (BCR) and other receptors, transitional B cells further differentiate into either follicular (FO) or marginal zone (MZ) B cells, now considered mature or naïve B cells [39, 43]. After specific antigen-recognition via BCR, selected B cells become either antibody-producing plasmablasts or plasma cells, or memory B cells [39]. Memory B cells are B cells, which circulate through the body [44]. Upon binding of the specific antigen, that originally activated the parent B cell that led to the production of the memory B cell, to the BCR, a strong and more rapid antibody response is initiated by the memory B cell [45]. In addition to the generation of immunological memory through memory B cells [45], B cells also contribute to immune responses through cytokine production [46], and antigen presentation, as well as through antibody production, which is their main contribution to allergy [47]. These multifaceted roles of B cells were also shown in several studies using allergic mouse models of asthma [48–50].

### Regulatory B cells (Bregs)

B cells can also regulate immune responses by other mechanism than antibody production, antigen presentation, and cytokine production. In the 1970s, allergen sensitization studies described an immunosuppressive potential of B cells for the first time [51, 52]. At that time, the precise mechanisms of suppression were not identified, and for more than 20 years, the study of immunosuppressive B cells did not receive significant attention [9, 53]. However, during the past two decades, the knowledge of regulatory B cell (Breg) phenotype and function increased [12]. In 2002, Mitzoguchi et al. described interleukin 10 (IL-10) producing B cells with regulatory functions that are characterized by CD1d upregulation in murine models of intestinal inflammation [54]. Other studies in murine models also showed an essential role of IL-10-producing B cells in controlling autoimmunity [55] and the prevention of arthritis [56]. Regulatory B cells (Bregs) were not only described in mice but they were also identified in humans: In 1998, Akdis et al. described IL-10-producing B cells in humans in the context of high-allergen exposure models and allergen immunotherapy (AIT). In AIT, high doses of the allergen are administered, which significantly changed IL-10 production after 7 days of bee venom (BV)-AIT in epitope-specific T cells, but not in B cells [57]. In 2007, Goetz et al. and Dass et al. demonstrated that therapy with Rituximab, an anti-CD20-antibody that depletes B cells, leads to severe exacerbation of ulcerative colitis and new onset of psoriasis in humans. Furthermore, they showed an association with the suppression of local IL-10 production suggesting an important anti-inflammatory role of IL-10-producing B cells in humans [58, 59].

#### Human Breg subsets

Several murine and human disease models were used to study the phenotype of Bregs as well as the underlying molecular mechanisms of Breg-mediated immune suppression. This resulted in the identification of different Breg subsets in both mice and humans with distinct phenotypic and functional properties [9]. Until now, there is no clear consensus on the classification and definition of Bregs. Here, we discuss the major Breg subsets that were identified in humans (Table 1).

**Table 1:** Different subsets and functions of human Bregs

Name	Phenotype	Functions
B10 cells	CD27^+^CD24^hi^	Regulate cytokine production by monocytes [60]
Immature transitional B cells	CD19^+^CD24^hi^CD38^hi^	Suppress T_H_1 and T_H_17 cell differentiation [15, 61] Induce conversion of Tregs [61] Regulate T cell immunity in CHB [62]
B_R_1 cells	CD19^+^CD25^+^CD71^+^CD73^−^	Suppress CD4^+^ T cell proliferation Production of IgG_4_ antibodies [63]
Plasmablasts	CD19^+^CD27^int^CD38^+^	Inhibit DC functions to generate autoreactive T cells [16]
Plasma cells	IgA^+^PD-L1^+^IL-10^+^	Suppress anti-tumour immunity [64]
GrB^+^ B cells	CD19^+^CD38^+^CD1d^+^IgM^+^CD147^+^	Suppress T cell proliferation by degradation of TCR [65]
CD9^+^ B cells	CD19^+^CD9^+^	Induction of T cell apoptosis [66]
CD5^+^CD1d^+^ B cells	CD19^+^CD5^+^CD1d^hi^	Suppress T_H_17 response [67] and IL-22 production [68]

Human Bregs are found among B-lymphocytes at different stages of maturation and differentiation: Human CD27^+^CD24^hi^ B10 cells produce IL-10 and thereby regulate cytokine production by monocytes after in vitro stimulation with lipopolysaccharide (LPS) and CpG, indicating a functional link between Bregs and the innate immune system. The mean frequencies of these cells are significantly increased in patients with autoimmune diseases [60]. Human CD19^+^CD24^hi^CD38^hi^ immature transitional B cells also have regulatory functions. After stimulation with CD40, these cells produce IL-10 thereby suppressing the differentiation of naïve T cells into T_H_1 and T_H_17 cells while inducing the conversion into Tregs [15, 61]. Moreover, this Breg subset was shown to regulate T cell immunity in chronic HBV (CHB) infection [62]. Human CD19^+^CD25^+^CD71^+^CD73^−^ B_R_1 cells are characterized by high expression of IL-10 and suppression of antigen-specific CD4^+^ T cell proliferation. Also, anti-inflammatory IgG_4_ antibody production was shown by these B_R_1 cells [63]. Lindner et al. described IL-21-induced granzyme B-expressing B cells (GrB^+^ B cells) that are found in the microenvironment of solid tumors next to IL-21-providing T cells. They are characterized by a CD19^+^CD38^+^CD1d^+^IgM^+^CD147^+^ expression signature and express regulatory molecules such as GrB, IL-10, CD25, and indolamine-2,3-dioxygenase (IDO). It was shown that they suppress T cell proliferation through GrB-dependent T cell receptor (TCR) degradation [65]. Another human Breg subset was described in 2017 by Brosseau et al. showing that CD9^+^ B cells induce effector T cell apoptosis in both mice and humans by IL-10 secretion. Moreover, they showed that these CD9^+^ B cells with regulatory properties are reduced in patients with severe asthma [66]. Human CD19^+^CD27^int^CD38^+^ plasmablasts produce IL-10 and secrete IgM. Interestingly, Matsumoto et al. showed that human CD19^+^CD27^int^CD38^+^ plasmablasts derived from naïve and especially immature B cells, but not human CD19^+^CD27^hi^CD38^+^ plasmablasts derived from memory B cells, are the major IL-10-producing B cells [16]. For human IgA^+^PD-L1^+^IL-10^+^ plasma cells, suppression of anti-tumor immunity was described [64]. Patients with tuberculosis were shown to have an increased number of CD5^+^Cd1d^+^ B cells with a stronger suppressive activity by inhibiting Th17 cell activation and IL-22 production [67, 68].

Due to this heterogeneity of Breg subsets, it was not possible until now to identify a Breg-specific transcription factor [9]. Since this is an emerging field of research, many questions still need to be answered with regard to plasticity, ontogeny, and the Breg-mediated suppression mechanism.

#### Several ways of Breg induction

The heterogeneity of Breg subsets leads to the assumption that there are either various distinct Breg lineages or that IL-10 can be induced by external stimuli at different stages of B cell development [9]. The precise mechanisms and required signals for Breg differentiation still need to be elucidated. However, several murine and human studies revealed that B cells of different maturational and differentiation stages are able to differentiate into Bregs upon antigen recognition and/or several kinds of stimuli [69–71]. CpG stimulation results in the generation of human CD19^+^CD27^int^CD38^+^ plasmablasts. Additionally treating B cells with IL-2, IL-6, and especially with the type I interferon IFN-α results in the generation of IL-10 secreting human CD19^+^CD27^int^CD38^+^ plasmablasts [16]. IFN-α was described to induce CD38^+^ expression on human naïve B cells [72] and promote differentiation into plasma cells [73]. In this regard, it is likely that IFN receptor signaling is required for IL-10-producing human plasmablasts [16]. Indeed, treatment with IFN-β, which is another type I IFN, increases IL-10 expression of human B cells after BCR and CD40 ligation [74]. Another study in mice revealed that stimulation with IL-1β, IL-6, and anti-CD40 in combination promotes the differentiation into IL-10-producing Bregs. Interestingly, B cell stimulation with TNF-α or IL-17 with or without anti-CD40 has no effect on IL-10 production [70]. It was also demonstrated that the TLR-MyD88-STAT3 pathway not only leads to antibody production of human B cells, but also regulates IL-10 production of human B cells by TLR7/8, which is enhanced by IFN-α [75]. The BATF/IRF-4/IRF-8-axis was also shown to play a role in IL-10 and IL-35 expression of murine regulatory B cells [76]. Matsumoto et al. also showed that IRF4 is required for the differentiation into IL-10-expressing murine plasmablasts, both in vitro and in vivo [16]. Moreover, IL-21 was described to induce IL-10 expression of murine B10 cells [71] and human GrB^+^ Bregs [65]. Also, in vitro stimulation of murine B cells with other molecules such as LPS, phorbol myristate acetate (PMA), and ionomycin generates IL-10-expressing B cells [77–79]. Menon et al. revealed that also plasmacytoid dendritic cells (pDCs) can drive the differentiation of human CD19^+^CD24^hi^CD38^hi^ Bregs and plasmablasts that express IL-10, IL-6, and TNF-α [69].

As another mechanism for Breg induction, overexpression of IL-10 in primary human B cells was shown to upregulate suppressor of cytokine signaling 3 (SOCS3), glycoprotein A repetitions predominant (GARP), IL-2 receptor α chain (CD25), and programmed cell death ligand 1 (PD-L1). Moreover, IL-10 overexpressing human B cells secrete less pro-inflammatory cytokines such as TNF-α or IL-8, whereas production of anti-inflammatory IL-1 receptor antagonists (IL-1RN) and vascular endothelial growth factor (VEGF) is increased [80]. In addition, the aryl hydrocarbon receptor (AhR) was shown to regulate the differentiation and function of IL-10-producing murine Bregs [81] and A proliferation-inducing ligand (APRIL) drives the differentiation of naïve human B cells to IL-10-producing Bregs [82]. Human type 3 innate lymphoid cells (ILC3s) were also shown to play a role in the induction of human IL-10-producing immature transitional Bregs [83], whereas IL-35 was described to induce IL-35-producing human Bregs (IL-35^+^ Bregs) through activation of STAT1/STAT3 [84].

#### Breg suppressor molecules

**Fig. 1:**
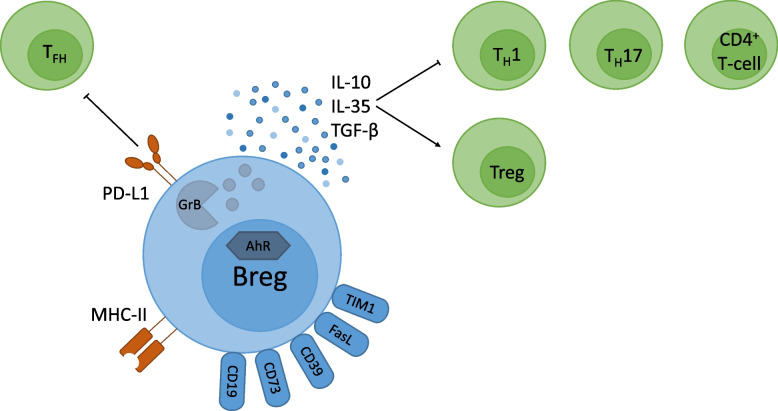
Breg suppressor molecules and their effect on other immune cells. Bregs mainly secrete the anti-inflammatory cytokines IL-10, IL-35, and TGF-β that in turn have a suppressive function on T_H_1 and T_H_17 cells. In addition, the conversion of CD4^+^ T cells into Tregs is induced. The transmembrane protein PD-L1 regulates cell expansion and differentiation of T_FH_ cells. Also, other surface-bound molecules such as MHC-II, CD19, CD73, CD39, FasL, and TIM1 as well as AhR and GrB are described to play a role in Breg-mediated immunosuppressive effects. IL, interleukin; TGF-β, transforming growth factor β; T_H_, T helper cell; Treg, regulatory T cell; PD-L1, programmed cell death ligand 1; T_FH_, follicular helper T cells, MHC-II, major histocompatibility complex II; FasL, Fas ligand; TIM1, T cell immunoglobulin and mucin domain 1; AhR, aryl hydrocarbon receptor; GrB, granzyme B

Regulatory B cells mainly act by secreting immune-modulatory cytokines. Although research mainly focused on the role of IL-10 as the hallmark cytokine of Bregs [54–56, 63, 85], three main cytokines were identified to be expressed by Bregs as suppressor molecules: IL-10, TGF-β [86], and IL-35 [87, 88] (Fig. 1). The anti-inflammatory cytokine IL-10 is involved in the maintenance of homeostasis by dampening inflammatory responses and can be produced by many cell types such as monocytes, T cells, DCs, NK cells, macrophages, mast cells, and B cells. It induces immune tolerance in patients with chronic inflammatory diseases such as allergy, autoimmunity, organ transplantation, and tumor tolerance and has direct and indirect suppressive effects on cytokine production and proliferation of effector T cells [89]. Moreover, IL-10 has an effect on B cells, including B cell survival, class-switch recombination, and plasma cell differentiation [25, 89]. IL-10 is also thought to have a positive effect on asthma pathophysiology by suppressing the IgE-mediated allergic cascade and decreasing airway inflammation [37]. Production of IL-10 was shown to be negatively regulated by B cell lymphoma-3 (Bcl-3) [90, 91], which is an atypical member of the inhibitor of NF-κB (IκB) protein family and regulates NF-κB-mediated gene expression, thereby regulating TLR signaling [92, 93]. TGF-β is an anti-inflammatory cytokine that has an important role in immune regulation, wound healing, and tissue remodeling and is involved in the conversion of naïve CD4^+^ T cells into functional regulatory T cells (Tregs) [25, 89]. IL-35 is primarily produced by Tregs and induces Treg proliferation while suppressing T_H_17 and T_H_1 responses [25, 84].

In addition to the expression of these anti-inflammatory cytokines, other anti-inflammatory molecules such as PD-L1 are associated with suppressive effects of Bregs. PD-L1, also known as CD274, is a transmembrane protein that regulates induced Treg cell function, development, and maintenance [94]. Bregs expressing PD-L1, described as PD-L1^hi^ B cells, regulate cell expansion and differentiation of follicular helper T cells (T_FH_), thereby suppressing autoimmune disease [95]. Also other surface-bound molecules like the Fas ligand (FasL) [96], CD39 [97], CD73 [97], CD19 [78], major histocompatibility complex II (MHC-II) [71], T cell immunoglobulin, and mucin domain 1 (TIM1) [98] as well as GrB [65], AhR [99], and intracellular signaling molecules such as STAT3 and MyD88 [100] are described to play a role in Breg-mediated immunosuppressive effects. This shows that there is a huge variety of suppressor molecules that are expressed by Bregs to fulfill their immunosuppressive function (Fig. 1).

### Role of Bregs in (childhood) allergic asthma

Recent research has concentrated on the role of Bregs in allergic asthma [26]. As mentioned above, Bregs express the anti-inflammatory cytokine IL-10, which is thought to have a positive effect on asthma pathophysiology by suppressing the IgE-mediated allergic cascade and decreasing airway inflammation [37]. Parasite infection in allergic mice models for asthma reveals protection from lung inflammation and airway hyperresponsiveness through IL-10-producing Bregs [37, 85]. Moreover, in human studies a higher number of allergen-specific, IL-10-producing Bregs is found upon allergen immunotherapies for cow milk and bee venom, indicating that higher Breg numbers are characteristic for induced tolerance to allergens [63, 101]. Braza et al. showed that upon allergen exposure the number of IL-10-producing Bregs is decreased in the lungs of asthmatic mice, indicating that the homeostasis of Bregs is altered by asthma [102]. Recently, Qian et al. showed that asthmatic patients have higher levels of IL-10 but lower levels of Bcl-3, suggesting that they have an important role in asthma pathogenesis. They also revealed that mice lacking Bcl-3 have increased eosinophilic airway inflammation, augmented airway goblet cell hyperplasia, elevated airway hyperresponsiveness, and increased levels of epithelial chemokines in the lungs after stimulation with HDM compared to control mice. These data demonstrate that Bcl-3 limits the IL-10 expression during allergic sensitization, thereby preventing lung inflammation and asthma pathogenesis in HDM-induced mice [103]. Since it was already shown that Bcl-3 is a negative regulator for IL-10 [91], these results indicate that Bcl-3 is a critical inhibitor of IL-10 in allergic asthma and targeting the Bcl-3/IL-10 axis may be a promising approach for allergic asthma therapy [103].

In adults, patients with allergic asthma show a lower percentage and absolute number of CD19^+^CD24^hi^CD27^+^ circulating Bregs [20, 21]. Also, these Bregs express less IL-10 upon LPS [104], but not CpG [105] stimulation. Moreover, frequencies of CD5^+^ and Cd1d^+^CD5^+^ Bregs are decreased in adult allergic asthmatics [22]. Recently, Sheehan et al. revealed that pediatric patients with allergic asthma also have significantly lower levels of circulating IL-10 expressing CD24^+^CD38^+^ Bregs, compared to the healthy control group. This could indicate that the lower Breg levels are associated with suboptimal control of allergic inflammation leading to asthma development or excess morbidity from asthma. To this end, it seems that Bregs are important for both children and adults with allergic asthma and that the appropriate number and function of Bregs may be mandatory to control asthma by releasing suppressive signals to decrease T_H_2 inflammation [23].

## Conclusion

During the past two decades, several Breg subsets were described in mice and humans, each with distinct phenotypical and functional characteristics. However, in contrast to regulatory T cells, which can be clearly defined by the expression of the transcription factor Foxp3, the identification of specific markers or transcription factors that clearly define Bregs is still missing. In addition, the precise mechanism and required signals for Breg differentiation are not completely understood. The way by which Bregs mainly exert their immunosuppressive function is through anti-inflammatory cytokines such as IL-10, TGF-β, and IL-35. Other anti-inflammatory molecules such as PD-L1, FasL, or intracellular signaling molecules like STAT3 and MyD88 are also associated with Breg function; however, this varies between different human Breg subsets. Several studies showed that IL-10 expression has a positive effect on asthma pathophysiology and that higher Breg numbers are characteristic of allergen tolerance. Moreover, it has been shown that asthma alters the homeostasis of Bregs: in adult as well as pediatric patients with allergic asthma, a lower percentage and absolute number of several IL-10 expressing Breg subsets are found. This suggests that lower Breg levels are associated with suboptimal control of allergic inflammation, which in turn may lead to the development of asthma. Since this is an emerging field of research, many questions still need to be answered with regard to plasticity, ontogeny, and Breg-mediated suppression mechanism as well as their exact role especially in childhood allergic asthma.

## Data Availability

Not applicable.

## Abbreviations

AA: Allergic asthma
AhR: Aryl hydrocarbon receptor
AIT: Allergen immunotherapy
APC: Antigen-presenting cell
APRIL: A proliferation-inducing ligand
BATF: Basic leucine zipper transcription factor
Bcl-3: B cell lymphoma 3
BCR: B cell receptor
Breg: Regulatory B cell
CD: Cluster of differentiation
CHB: Chronic HBV
DC: Dendritic cell
FasL: Fas ligand
FcεRI: High-affinity IgE receptor
FO: Follicular
GARP: Glycoprotein A repetitions predominant
GrB: Granzyme B
HDM: House dust mite
IDO: Indolamine-2,3-dioxygenase
IFN: Interferon
Ig: Immunoglobulin
IgE: Immunoglobulin E
IκB: Inhibitor of NF-κB
IL: Interleukin
IL-1RN: IL-1 receptor antagonist
ILC: Innate lymphoid cell
ILC3: Type 3 innate lymphoid cell
IRF: Immunoregulatory factor
LPS: Lipopolysaccharide
MHC-II: Major histocompatibility complex II
MyD88: Myeloid differentiation primary response 88
MZ: Marginal zone
NF-κB: Nuclear factor kappa-light-chain-enhancer of activated B cells
NK: Natural killer
PD-L1: Programmed cell death ligand 1
pDCs: Plasmacytoid dendritic cells
PMA: Phorbol myristate acetate
SOCS3: Suppressor of cytokine signaling 3
STAT: Signal transducer and activator of transcription
TCR: T cell receptor
T_FH_: Follicular helper T cells
TGF-β: Transforming growth factor β
T_H_: T helper cell
TIM1: T cell immunoglobulin and mucin domain 1
TLR: Toll-like receptor
TNF: Tumor necrosis factor
Treg: Regulatory T cell
VEGF: Vascular endothelial growth factor
WHO: World Health Organization
